# The Effects of Two Different Concurrent Training Configurations on Markers of Metabolic Syndrome and Fitness in Women With Severe/Morbid Obesity: A Randomised Controlled Trial

**DOI:** 10.3389/fphys.2021.694798

**Published:** 2021-09-21

**Authors:** Pedro Delgado-Floody, Alberto Soriano-Maldonado, Manuel A. Rodríguez-Pérez, Pedro Ángel Latorre-Román, Cristian Martínez-Salazar, Claudia Andrea Vargas, Felipe Caamaño-Navarrete, Daniel Jerez-Mayorga, Cristian Álvarez

**Affiliations:** ^1^Department of Physical Education, Sport and Recreation, Universidad de La Frontera, Temuco, Chile; ^2^Department of Education, Faculty of Education Sciences, University of Almería, Almería, Spain; ^3^SPORT Research Group (CTS-1024), CERNEP Research Center, University of Almería, Almería, Spain; ^4^Department of Didactics of Corporal Expression, University of Jaen, Jaen, Spain; ^5^Faculty of Education, Universidad Católica de Temuco, Temuco, Chile; ^6^Faculty of Rehabilitation Sciences, Universidad Andres Bello, Santiago, Chile; ^7^Department of Health, Universidad de LosLagos, Osorno, Chile

**Keywords:** concurrent training, morbid obesity, exercise training, metabolic syndrome, exercise order, interindividual variability

## Abstract

Concurrent training (CT), characterised by combining both aerobic and resistance training modalities within the same session, is recognised to improve metabolic syndrome (MetS) markers, but little is known about the effects of different configurations (i.e., order) of these exercise modalities on MetS markers and the interindividual responses. The purpose of the present study was to describe the effects, and the interindividual variability, of 20weeks of two CT configurations (i.e., high intensity interval training (HIIT) plus resistance training (RT), compared with RT plus HIIT) in women with severe/morbid obesity. Overall, 26 women with severe/morbid obesity were assigned either to HIIT+RT [*n*=14, mean and 95%CI, 45.79 (40.74; 50.83) or RT+HIIT (*n*=12), 33.6 (25.30; 41.79) years]. MetS-related outcomes were waist circumference (WC, cm), systolic (SBP, mmHg) and diastolic (DBP, mmHg) blood pressure, high-density lipoprotein cholesterol (HDL-c), triglycerides (Tg), and fasting plasma glucose (FPG). Secondary outcomes were other anthropometrics, body composition, lipids, muscle strength, and the six-minute walk test (6Mwt). There were significant differences in the prevalence of nonresponders (NRs) only for WC comparing HIIT+RT 2 (18.1%) vs. RT+HIIT group 5 (50.0%), *p*<0.0001, but not for SBP 4 (27.2%) vs. 4 (40.0%), DBP 8 (72.7%) vs. 7 (70.0%), FPG 8 (72.7%) vs. 9 (90.0%), HDL-c 7 (63.6%) vs. 8 (80.0%), and Tg 7 (63.6%) vs. 8 (80.0%), all *p*>0.05. Additionally, the RT+HIIT group showed significant reductions in WC (∆ –3.84cm, *p*=0.015), SBP (∆ –8.46mmHg, *p*=0.040), whereas the HIIT+RT group elicited significant reductions only in SBP (∆ –8.43mmHg, *p*=0.022). The HIIT+RT promoted a lower prevalence of NRs than the RT+HIIT configuration on WC, and overall, there were slightly more beneficial training-induced effects on markers of MetS in the RT+HIIT group compared to the HIIT+RT group.

## Introduction

Morbid obesity, defined as a body mass index (BMI) of ≥40kg/m^2^ (class III obesity), is a chronic disease with life-threatening cardiometabolic consequences such as elevated blood pressure [systolic (SBP) or diastolic BP (DBP)], fasting plasma glucose (FPG), triglycerides (Tg), and low high-density lipoprotein cholesterol (HDL-c), all summarised as metabolic syndrome (MetS; Baffi et al., 2016), substantially increasing the rates of total mortality, with most of the excess deaths due to heart disease, cancer, diabetes, and important life expectancy reductions compared with normal weight peers (Kitahara et al., 2014). Moreover, morbid obesity has been associated with impairments of cardiorespiratory fitness and muscle strength, limiting the capacity to perform activities of daily living (Pazzianotto-Forti et al., 2020). Additionally, this fact increases the economic costs associated with healthcare in this population (Espallardo et al., 2017).

Due to the multi-factorial aetiology of morbid obesity, such as the genetic load (e.g., the *FTO* gen), and other environmental factors, including mainly lifestyle determinants such as physical activity, exercise training participation, and diet as the main modulators, the application of lifestyle strategies have been proposed prior to alternative surgical intervention in these populations (Bächler et al., 2013; Espallardo et al., 2017). In this sense, exercise training such as the resistance training (RT), defined as any exercise that causes voluntary skeletal muscle contraction by using external weights including dumbbells and metal bars (Schoenfeld et al., 2017), is a known non-pharmacotherapy strategy for improving muscle strength and functional capacity in obese patients undergoing bariatric surgery (Huck, 2015). Similarly, high intensity interval training (HIIT), defined as several and brief bouts of high-intensity effort usually by cycling/running, interspersed by recovery periods (Gibala et al., 2012; Delgado-Floody et al., 2020), has produced strong evidence for the improvement of cardiometabolic risk factors for type 2 diabetes mellitus, arterial hypertension, central arterial stiffness, vascular function, and cardiorespiratory fitness (Ramírez-Vélez et al., 2019). Thus, HIIT might have protective effects against the development of cardiometabolic diseases including populations with poor glucose control and high blood pressure in comparison to moderate-intensity continuous training (MICT; Campbell et al., 2019). Thus, following RT or HIIT alone, unique physiological adaptations have been described for improving, for example, muscle strength, the endurance performance by walking test, as well as beneficial metabolic improvements at MetS markers including fasting glucose reductions, increases at HDL-c, and decreases in Tg in populations with higher adiposity (de Matos et al., 2018).

Thus, in individuals with morbid obesity for example, exercise training has proven to be effective for inducing clinically significant weight loss (5–10%; Gerber et al., 2015), and for the reduction of cardiovascular risk (Delgado-Floody et al., 2019), in accordance with the standard recommendations for these cohorts prior to bariatric surgery. Additionally, there are also other benefits such as the increase of skeletal muscle mass, the reduction of body fat, and better glucose control by the lowering of FPG, and lipids regulation (i.e., increases of HDL-c, and decreases of Tg; Han and Lean, 2016). Briefly, 12weeks of concurrent training (CT), defined as a combination of both MICT/plus RT, decreased body weight (by ~7.3kg), blood pressure, and FPG in this cohort (Marc-Hernández et al., 2019). In addition, part of our preliminary findings have shown that 20weeks of RT decreases MetS risk factors in morbidly obese patients, showing a low inter-individual variability in those patients with greater adiposity, revealing that with more adiposity alteration, the benefits of RT are also more visible (Delgado-Floody et al., 2019). However, some inconsistences, which are directly related with the ‘order’ (i.e., starting the CT session with MICT followed by RT, or vice versa) of the CT session, have been described after CT. For example, some literature reports that by starting the CT exercise session by RT, participants can get more benefits and improve their physical fitness markers (i.e., increases of upper body strength and neuromuscular markers; Murlasits et al., 2018). However, in contrast, other studies have reported that starting CT with MICT/or HIIT does not alter physiological adaptations in similar outcomes (Wilhelm et al., 2014). Other evidence shows that starting CT with RT exercises first clearly promotes greater lower-body strength gains and neuromuscular economy (Cadore et al., 2013). By contrast, other reports claim no more benefits by starting with MICT/HIIT or RT to physical fitness markers in populations of athletes (Eddens et al., 2018), and additionally, no other benefits for decreasing body fat using one or another exercise modality when starting the CT session (Cadore et al., 2012). However, little is known about the interindividual variability to exercise training (IVET) in relation to different orders of sessions of CT in morbidly obese populations, and in health-related outcomes, such as MetS markers. Briefly, IVET means that some subjects achieve benefits after training, and are termed responders (Rs), while others exhibit a worsened or unchanged response, that are commonly known as nonresponders (NRs; Bouchard et al., 2012). With regard to the causes of IVET, genetic (Stephens et al., 2015) and environmental factors, including the mode of exercise training (Alvarez et al., 2017), have been described. In addition, considering the health-related benefits of CT including MICT/or HIIT plus RT in terms of physical fitness, and the metabolic markers in different populations, as well as taking into account the previous discrepancies of the order session in relevant literature, it is necessary to investigate the exercise modalities interaction as a precision medicine for improving MetS markers. The purpose of the present study was to describe the effects, and the interindividual variability of 20weeks CT in different orders by HIIT plus RT compared with another group doing RT plus HIIT in women with severe/morbid obesity at risk of MetS.

## Materials and Methods

### Study Design and Participants

This study is a parallel-group randomised controlled trial in which 34 women with morbid obesity were randomly allocated to one of the two similar CT exercise programmes. The exercises were then applied in different session orders by the two groups (HIIT+RT, *n*=17), and resistance training plus high-intensity interval training (RT+HIIT, *n*=17). The sample size was calculated by G*Power software, and by using the observed delta changes in FPG after previous CT exercise interventions of −4.0mg/dl, and a standard deviation of 1.0mg/dl (Álvarez et al., 2019). Thus, a sample with a minimum of four cases per group (minimum sample of *n*=8), gave us an alpha error of *α*=0.05, and a *β*=0.80. All participants were informed of the pre–post procedures and of the possible risks/benefits potentially involved with participation in the study, after which they signed an informed consent. The study follows the CONSORT guidelines for randomised trials, was developed in accordance with the Declaration of Helsinki (2013), and has been approved by the Ethical Committee of the Universidad de La Frontera, Temuco, Chile (DI18-0043 Project).

**Figure 1:**
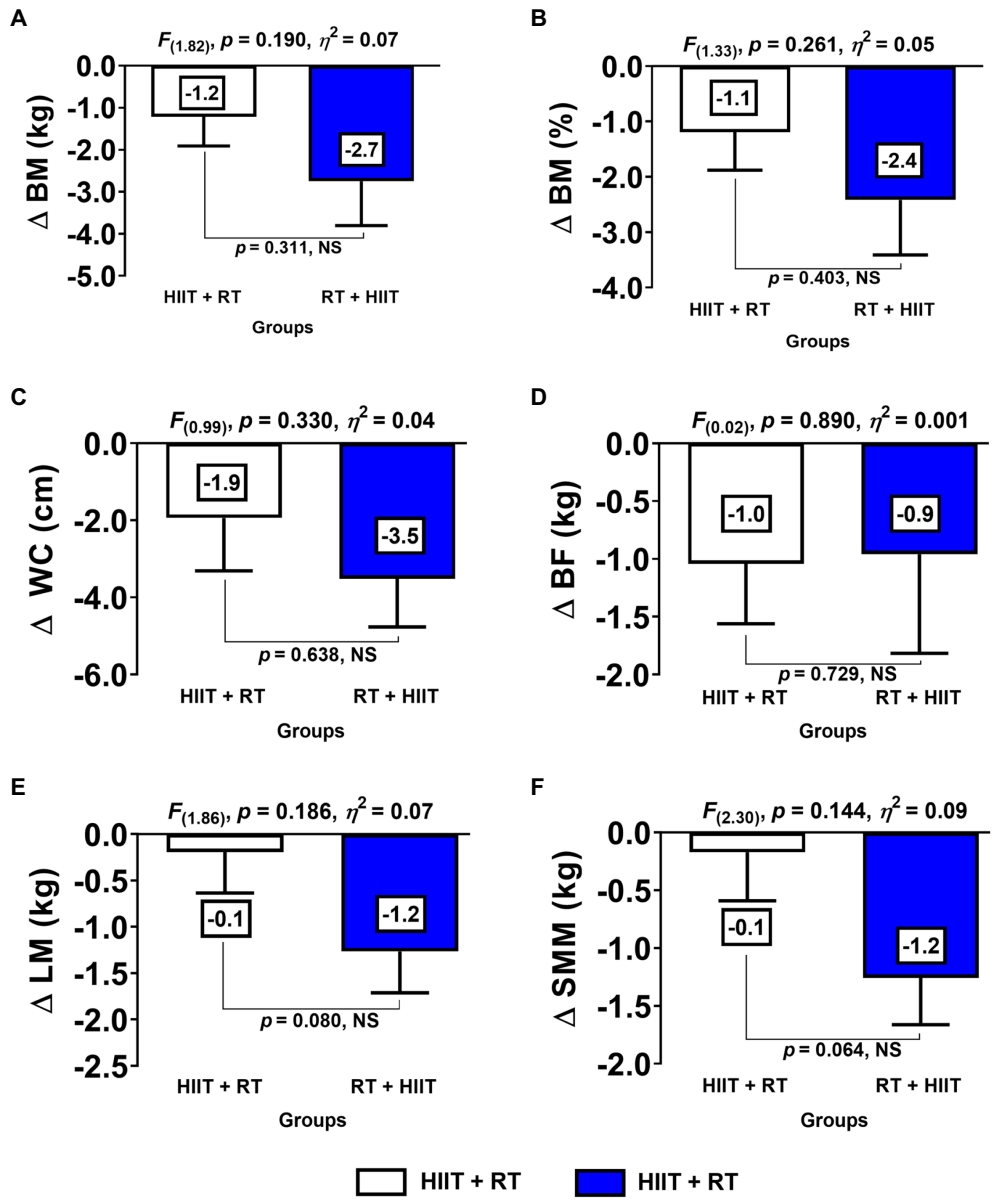
Changes in body composition and anthropometric parameters for each experimental exercise group. (HIIT+RT) High-intensity interval training plus resistance training order group. (RT+HIIT) Resistance training plus high-intensity interval training order group. (BM) body mass (BF) body fat (SMM) skeletal muscle mass (LM) lean mass (WC) waist circumference. (∆) denotes delta changes from pre–post intervention. (NS) denotes no significant differences.

Eligibility criteria were as follows; (i) being a candidate for bariatric surgery (ii) aged between 18 and 60years (iii) being medically authorised, and (iii) with a BMI ≥40kg/m^2^ or ≥35kg/m^2^ with additional comorbidities (i.e., diabetes, hypertension, insulin resistance) controlled by pharmacotherapy according to the Chilean requirements for morbidly obese patients in order to be a candidate for bariatric surgery (Carrasco et al., 2005). Exclusion criteria were; (i) having physical limitations preventing the performance of exercise (e.g., restricting injuries of the musculoskeletal system) (ii) having exercise-related dyspnoea or respiratory alterations (iii) having chronic heart disease with any worsening in the last month, and (iv) adhering to less than 80% of the total interventions (these results were excluded from the statistical analyses). After the enrolment stage, 43 (*n*=43) participants were assessed for eligibility and nine (*n*=9) were not included according to the inclusion criteria. After the lost followed up participants (*n*=8) for data analysis, 26 (*n*=26) participants were part of the final sample size as follows; HIIT+RT group [*n*=14, mean and (95%CI); 45.79 (40.74; 50.83) or to RT+HIIT group (*n*=12), 33.6 (25.30; 41.79) years old]. Clinical trial number registration is NCT04932642. The study design is shown in (Supplemetary Figure 1).

### Interindividual Variability With Regard to Concurrent Exercise Training

Following previous criteria applied in exercise training interventions (Bouchard et al., 2012), the IVET was categorised as responders (Rs), and nonresponders (NRs), using the typical error measurement (TE). Thus, we used previous TE×2 calculated for WC (0.50cm×2), SBP (4.01mmHg×2), DBP (2.49mmHg×2), HDL-c (2.5mg/dl×2), Tg (12.3mg/dl×2), using the known equation: $\mathrm{TE}=D_{diff}/\sqrt{2}$, where *SD_diff_* is the variance (standard deviation) of the difference scores observed between the two repetitions of each test. The NRs for all the MetS outcomes were defined as those individuals who failed to demonstrate a decrease or increase (in favour of beneficial changes) that was greater than twice the TE away from zero.

### Metabolic Syndrome Markers

The MetS markers were screened using standard criteria (Alberti et al., 2009). All participants were instructed to arrive at the laboratory with an overnight fasting of 8–10h, being measured between 08:00 and 9:00 in the morning. These conditions were taken at baseline (pre-test), and at post intervention (post-test). Blood samples were taken with an extraction of ~5ml, in order to determine the MetS outcomes; FPG, HDL-c, and triglycerides (Tg), as well as the additional markers, total cholesterol (Tc), and low-density lipoprotein cholesterol (LDL-c). Briefly, the samples were placed on ice and centrifuged at 2000×*g* for 5min at 4°C. Plasma samples were immediately transferred to pre-chilled microtubes and stored at −20°C for the following analysis. The measurement of plasma glucose, total cholesterol, and triglycerides were analyzed by enzymatically standard kits (Wiener Lab, Inc., Rosario, Argentina) using automatic equipment (Metrolab2300 Plus^™^, Metrolab Biomed, Inc., Buenos Aires, Argentina). The HDL-c was analyzed by enzymatic methods after phosphotungstate precipitation (Demacker et al., 1997), and LDL-c was estimated by the Friedewald formula (Friedewald et al., 1972).

The SBP and DBP measurements were carried out according to the standard criteria (Mancia et al., 2014). Blood pressure was measured in the sitting position after 5min of rest. Two recordings were made, and the mean of the measurements was used for statistical analysis with an OMRON^™^ digital electronic BP monitor (model HEM 7114, Chicago, IL, United States). Previously to this measurement, we asked the participants to not smoke, or drink meals at least 30min prior to measurement. Waist circumference (WC) was assessed with a tape measure calibrated in centimetres (Adult SECA^™^, United States) at the upper hipbone and the top of the right iliac crest, with a non-elastic measuring tape in a horizontal plane around the abdomen at the level of the iliac crest. The tape was snug, but did not compress the skin and was parallel to the floor. The measurement was made at the end of a normal expiration (National Institutes of Health et al., 2000).

### Body Composition and Anthropometrics Parameters

The body composition and anthropometrics parameters were measured after fasting (>8h). Body mass (kg), and body fat (% and kg), skeletal muscle mass (kg), and lean mass (kg) were measured using a digital bio-impedance scale (TANITA^™^, model 331, Tokyo, Japan) and height (m) was measured using a SECA^™^ stadiometer (model 214, Hamburg, Germany), with subjects in light clothing and without shoes. BMI was calculated as the body mass divided by the square of the height (kg/m^2^). The BMI was determined to estimate the degree of obesity (kg/m^2^) using the standard criteria for obesity and severe/morbid obesity classification (Sturm, 2007).

### Six-Minutes Walking Test

The day after the metabolic measurements, the physical condition of the participants in both groups was measured by endurance and muscle strength testing. First, a six-minute walking test (6Mwt) was used to estimate CRF. The test was performed in a closed space on a flat surface (30m long), with two reflective cones placed at the ends to indicate the distance. During the test, participants were assisted with instructions from an exercise physiologist (de Souza et al., 2009).

### Handgrip Strength

Handgrip strength (HGS) was assessed using a digital dynamometer (Baseline^™^ Hydraulic Hand Dynamometers, United States), which has been used in previous studies (Norman et al., 2011). Two attempts were made, measuring each hand, and the best result from each was selected. As previously, the mean value obtained was taken as the total score (Norman et al., 2011). Using this data we calculated other outcomes such as the HGS and its variation ratio by body mass [HGS/BM], skeletal muscle [HGS/SMM], and lean mass [HGS/LM].

### Concurrent Training Intervention

The CT programme had two sections of HIIT and RT, which were applied in different orders to the two experimental groups; HIIT+RT, and RT+HIIT. Before the starting of each exercise-group, both HIIT+RT, and RT+HIIT participated in four familiarization sessions that included (i) knowledge of all measurements, exercise-machine, and instructions during the exercise program (ii) exercising in cycling, weights, and metal bars (iii) applying a few of the exercises of HIIT in 2–3 intervals, and RT in 2–3 sets of exercises, in order to know the configuration of each exercise, and (iv) applying 50–70% of their CT program, independent of their group. Following this, first, in the HIIT+RT group, the HIIT section consisted of 60s of maximum intensity exercise using a magnetic resistance static bicycle (Oxford^™^ Fitness, model BE-2701, Chile), followed by 60–120s of passive recovery with the bicycle totally off. This was repeated four to seven times according to the weekly schedule (Delgado-Floody et al., 2019). The intensity of the exercise was measured on the Borg scale of 1–10 of perceived exertion and the participants worked at a level of between 6 and 9 points.

Second, in the RT section, three out of four RT exercises were included (according to the planning week), targeting the following different muscle groups: (1) forearm (2) knee flexors and extensors (3) trunk (4) chest (5) shoulder elevators (6) horizontal shoulder flexors (7) extensors, and finally (8) plantar flexors. These exercises were performed in three sets of as many repetitions (continuous concentric/eccentric voluntary contraction) as possible in 60s, followed by 60–120s of passive recovery, as previously reported (Álvarez et al., 2019). To estimate the intensity of work in the different RT exercise, the maximum dynamic muscular strength (1RM) was estimated indirectly through the Brzycki formula (Brzycki, 1993), with fewer than 12 maximum repetitions. The RT+HIIT group performed the same training programme as the HIIT+RT group (described above) but the order of the HIIT and RT exercises were reversed (i.e., first RT and then HIIT). This was called the RT+HIIT group. The exercise programme compounds of the CT regime applied can be found in Table 1.

**Table 1:** Characteristics of the high-intensity interval training, and of the resistance training scheme, both compounds of a concurrent training programme of 20weeks, applied to two different groups; CT applied in order HIIT+RT, and CT applied in order RT+HIIT.

Concurrent training (*)												
Weeks	HIIT					RT						
	E N°	Intensity (Borg subjective perception)	Time (min)	Sets	Rest (min)	E N°	Intensity (%1RM)	Reps	Time (min)	Sets	Rest (min)	Duration (min)
1–2	3	6–7	1	4	2.0	3	40	20–25	1	3	2.0	45
3–4	3	6–7	1	4	2.0	3	40–50	25–30	1	3	2.0	45
5–6	4	6–7	1	5	1.5	4	45–50	30	1	3	1.5	45
7–8	4	7–8	1	5	1.5	4	45–50	30–35	1	3	1.5	45
9–10	4	7–8	1	5	1.5	4	50–55	25–30	1	3	1.5	45
11–12	4	7–8	1	6	1.5	4	50–55	25–30	1	3	1.5	45
13–14	4	7–8	1	6	1.0	4	50–55	30	1	3	1.0	45
15–16	4	8	1	6	1.0	4	50–55	30–35	1	3	1.0	45
17–18	4	8	1	7	1.0	4	55	30–35	1	3	1.0	45
19–20	4	8–9	1	7	1.0	4	55–60	30–35	1	3	1.0	45

(*) The present HIIT and RT dosage, components of the CT scheme, are valid to both experimental HIIT+RT, and RT+HIIT groups. (HIIT) High-intensity interval training. (RT) Resistance training. (E N°) number of intervals (HIIT groups) or number of total exercises (RT group).

### Statistical Analyses

**Figure 2:**
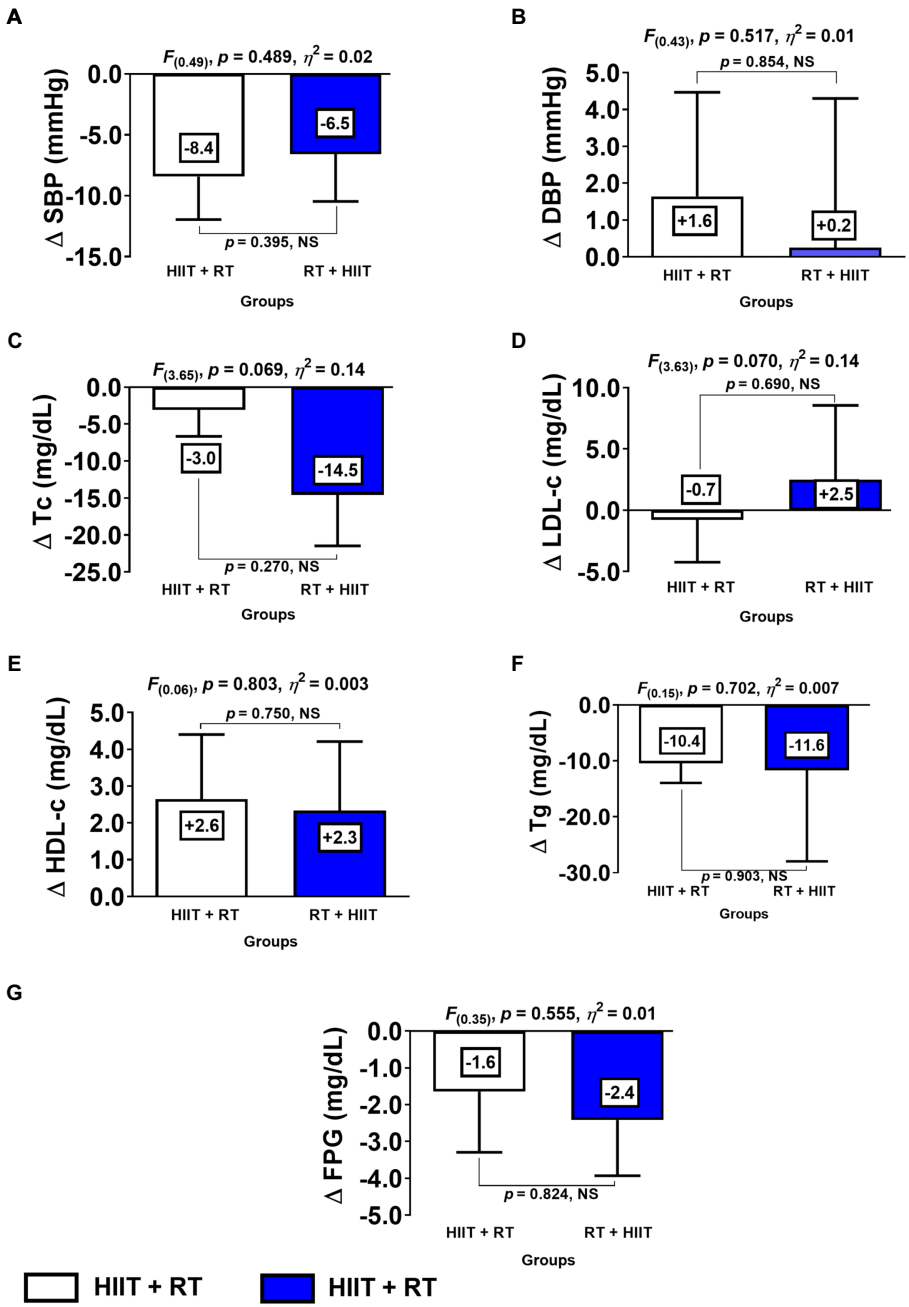
Changes of metabolic syndrome makers for each experimental exercise-group. (HIIT+RT) High-intensity interval training plus resistance training order group. (RT+HIIT) Resistance training plus high-intensity interval training order group. (SBP) systolic blood pressure (DBP) diastolic blood pressure (FPG) fasting plasma glucose (Tc) total cholesterol (LDL-c) low-density lipids (HDL-c) high-density lipids, and (Tg) triglycerides. (∆) denotes delta changes from pre to post intervention. (NS) denotes no significant differences.

**Figure 3:**
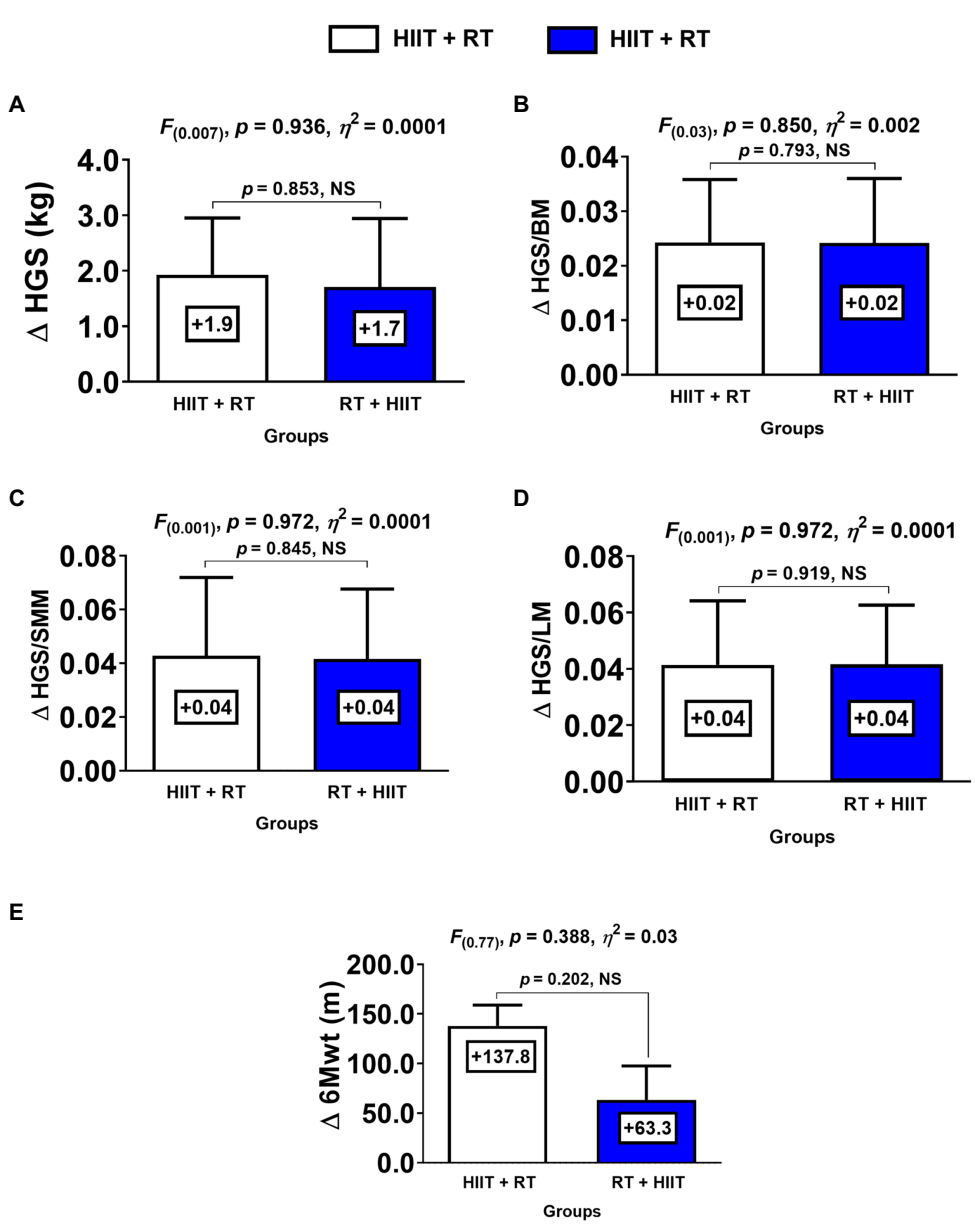
Changes in physical fitness for each group. (HIIT+RT) High-intensity interval training plus resistance training order group. (RT+HIIT) Resistance training plus high-intensity interval training order group. (HGS) handgrip strength (BM) body mass (SMM) skeletal muscle mass (LM) lean mass (6Mwt) six-minute walking test. (∆) denotes delta changes from pre to post intervention. (NS) denotes no significant differences.

**Figure 4:**
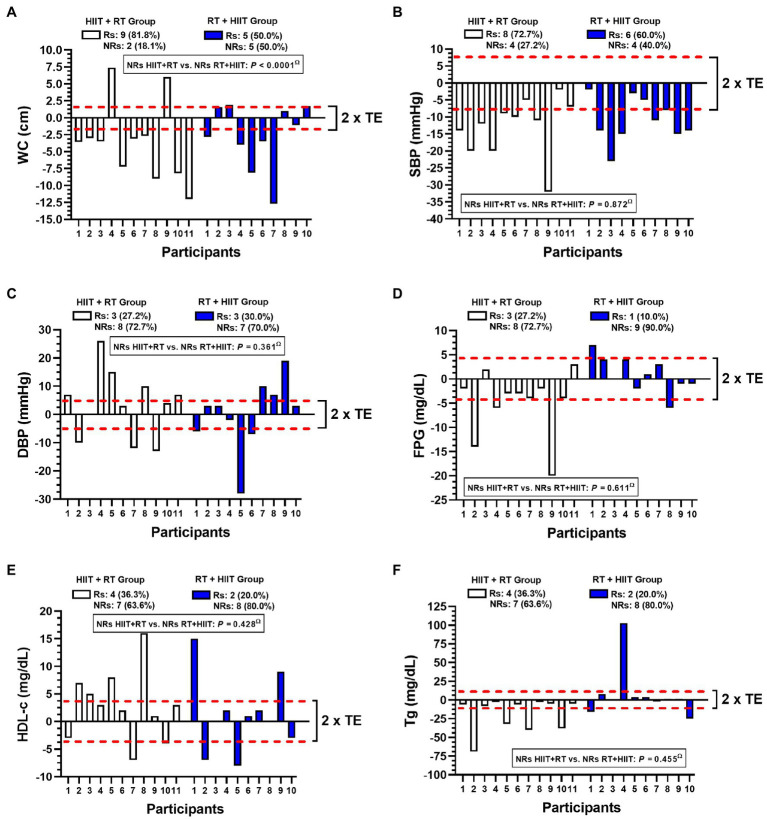
Inter-individual variability for a different concurrent training order session in morbid obesity patients in MetS outcomes. Abbreviations: WC; waist circumference; SBP, systolic blood pressure; DBP, diastolic blood pressure; FPG, fasting plasma glucose; HDL-c high-density lipids; and Tg, triglycerides. (Rs) denotes ‘responders’. (NRs) denotes non-responders to improve MetS outcomes considering modifications more than 2 technical errors. Bold values denote significant differences among frequencies of NRs between HIIT+RT vs. RT+HIIT group.

Data are presented as the mean and (95%CI) in tables, as mean with (±) standard error in Figures 1–3, and as individual delta in Figure 4 for identification of Rs and NRs. Normality and homoscedasticity assumptions for all data were analyzed using the Shapiro–Wilk and Levene’s test, respectively. In the HIIT+RT group, the Tg outcome, as well as in the RT+HIIT group, LDL-c, and lean mass outcomes were analyzed by the *Wilcoxon* non-parametric test. For training-induced changes, the student’s *t*-test was used to identify differences at baseline, while a repeated measure two-way ANOVA was applied to assess the occurrence of an actual training effect; namely, *p*<0.05 for the interaction (time×group) on the main MetS (WC, SBP, DBP, FPG, HDL, and Tg, as well as to the secondary outcomes). A Sidack’s *post hoc* test was used for multiple comparisons. Additionally, the Eta partial squared for interaction (Time×Group) was assessed by *η*^2^ obtained from the ANCOVA with small (*η*^2^=0.01), medium (*η*^2^=0.06), and large (*η*^2^=0.14) effects defined according to Lakens (2013). The prevalence of NRs was described using the comparisons by percentage between both experimental groups using a Chi square test *χ*^2^. All statistical analyses were performed with SPSS statistical software version 23.0 (SPSS^™^ Inc., Chicago, IL). The alpha level was set at *p*<0.05 for statistical significance.

## Results

### Baseline Characteristics

There were significant differences between the groups (*p*=0.004) in terms of age with 45.79 (95% CI; 40.74 to 50.83) in the HIIT+RT vs. 33.55 (95% CI; 25.30 to 41.79) in the RT+HIIT group. There were no other differences at baseline (Table 2).

**Table 2:** Training-induced changes on anthropometric and body composition in morbid obesity patients after 16weeks of concurrent training applied in two different concurrent training session orders; HIIT + RT, or CT applied as RT+HIIT.

Outcomes	Time	Groups		Baseline †*p* value
		HIIT + RT	RT+HIIT	
(*n* =)				
Age (y)		45.79 (40.74; 50.83)	33.55 (25.30; 41.79)	***p*=0.004**
Height (m)		1.55 (1.51; 1.59)	1.59 (1.55; 1.62)	*p*=0.188
**Anthropometric/Body composition outcomes**				
Body mass (kg)	Pre	99.30 (87.45; 111.15)	108.12 (99.84; 116.40)	*p*=0.109
	Post	98.09 (86.24; 109.93)	105.64 (97.05; 114.22)	
	*p* =	*p*=0.160	***p*=0.015**	
Body mass index (kg/m^2^)	Pre	40.95 (37.00; 44.89)	42.72 (39.77; 45.63)	*p*=0.217
	Post	40.23 (36.49; 44.01)	41.87 (38.73; 45.01)	
	*p* =	*p*=0.065	***p*=0.050**	
Waist circumference (cm)	Pre	114.22 (106.24; 122.18)	118.67 (110.43; 126.91)	*p*=0.246
	Post	112.27 (104.31; 120.23)	114.83 (106.31; 123.35)	
	*p* =	*p*=0.147	***p*=0.015**	
Body fat (%)	Pre	46.95 (43.72; 50.19)	48.95 (47.27; 50.63)	*p*=0.161
	Post	46.65 (43.90; 49.40)	49.05 (47.00; 51.10)	
	*p* =	*p*=0.413	*p*=0.807	
Body fat (kg)	Pre	47.59 (39.17; 56.01)	52.85 (47.49; 58.21)	*p*=0.143
	Post	46.55 (38.58; 54.51)	52.21 (46.04; 58.39)	
	*p* =	*p*=0.118	*p*=0.388	
Skeletal muscle mass (kg)	Pre	49.08 (45.54; 52.62)	52.21 (49.06; 55.37)	*p*=0.093
	Post	49.91 (45.02; 52.79)	51.00 (48.53; 53.48)	
	*p* =	*p*=0.677	***p*=0.015**	
Lean mass (kg)	Pre	51.70 (47.98; 55.42)	54.99 (51.66; 58.31)	*p*=0.094
	Post	51.51 (47.42; 55.60)	53.78 (51.18; 56.38)	
	*p* =	*p*=0.622	*p*=0.081^¥^	

*Data are shown as mean and (95%CI). Groups are described as; (HIIT+RT) order session of high-intensity interval training plus resistance training, (RT+HIIT) order session of resistance training plus high-intensity interval training. (†) Between-group baseline comparisons were measured by a General Lineal Model, using a Univariant test. Within-group comparisons by pre-post time were analyzed by Repeated Measures using 2-way ANOVA (Group×Time). (¥) Analyzed by non-parametric Wilcoxon test. Bold values denote significant differences at p≤0.05. All analyses were adjusted by age, height, and BMI*.

### Training-Induced Changes on Mets Markers and Secondary Outcomes

With regard to anthropometric and body composition and considering the absolutes values, in the RT+HIIT group there were significant changes between pre- and post-training measurements in outcomes relating to BMI: 42.72 (39.77; 45.63) vs. 41.87 (38.73; 45.01) kg/m^2^, *p*=0.050, WC: 118.67 (110.43; 126.91) vs. 114.83 (106.31; 123.35) cm, *p*=0.015, of skeletal muscle mass 52.21 (49.06; 55.37) vs. 51.00 (48.53; 53.48) kg, *p*=0.015, and the lean mass of this group: 54.99 (51.66; 58.31) vs. 53.78 (51.18; 56.38) kg, *p*=0.022 (Table 2). There were no other changes of the RT+HIIT or HIIT+RT groups (Table 2).

Comparing the HIIT+RT and RT+HIIT groups with regard to delta (∆) changes from pre to post test, there were no significant differences between groups at ∆BM kg, ∆BF%, ∆WC, ∆BF kg, ∆LM, and ∆SMM (Figures 1A–F).

With regard to cardiovascular and metabolic outcomes, and considering the absolutes values, there were significant changes in the HIIT+RT group from pre- to post-training measurements in SBP: 138.57 (128.50; 148.64) vs. 130.14 (123.20; 137.08) mmHg, *p*=0.022, to pre and post-test, respectively, (Table 3). No other pre- or post-training changes were observed in other outcomes in this group at these parameters. Similarly, for the RT+HIIT group, there were significant training-induced changes of SBP from 136.36 (125.74; 146.98) vs. 129.90 (122.85; 132.96) mmHg, *p*=0.040, and of total cholesterol from 177.09 (161.31; 192.87) vs. 162.09 (148.27; 175.90) mg/dl, *p*=0.017 (Table 3). No other pre- or post-training changes were observed in other outcomes in this group at these parameters.

**Table 3:** Training-induced changes on cardiovascular and plasma markers in morbid obesity patients after 20weeks of concurrent training applied in two different order session; CT as HIIT + RT, or CT applied as RT+HIIT.

Outcomes	Time	Groups		Baseline ^†^*p* value
		HIIT + RT	RT+HIIT	
**Cardiovascular/metabolic outcomes**				
Systolic blood pressure (mmHg)	Pre	138.57 (128.50; 148.64)	136.36 (125.74; 146.98)	*p*=0.741
	Post	130.14 (123.20; 137.08)	127.90 (122.85; 132.96)	
	*p* =	***p*=0.022**	***p*=0.040**	
Diastolic blood pressure (mmHg)	Pre	83.57 (77.02; 90.11)	91.54 (83.40; 99.68)	*p*=0.085
	Post	85.21 (80.74; 89.68)	90.27 (85.01; 95.52)	
	*p* =	*p*=0.614	*p*=0.728	
Fasting plasma glucose |(mg/dl)	Pre	99.71 (86.51; 112.91)	97.45 (86.62; 106.88)	*p*=0.961
	Post	98.07 (87.73; 108.40)	95.18 (88.00; 102.35)	
	*p* =	*p*=0.307	*p*=0.213	
Total cholesterol (mg/dl)	Pre	181.35 (163.65; 199.06)	177.09 (161.31; 192.87)	*p*=0.817
	Post	178.28 (157.47; 199.10)	162.09 (148.27; 175.90)	
	*p* =	*p*=0.558	***p*=0.017**	
Low-density lipoprotein cholesterol (mg/dl)	Pre	124.78 (111.02; 138.54)	101.18 (78.09; 124.26)	*p*=0.068
	Post	124.00 (110.93; 137.06)	104.09 (90.35; 117.82)	
	*p* =	*p*=0.868	*p*=0.509^¥^	
High-density lipoprotein cholesterol (mg/dl)	Pre	54.71 (47.89; 61.52)	49.18 (43.44; 54.92)	*p*=0.151
	Post	57.35 (50.84; 63.86)	51.45 (45.78; 57.12)	
	*p* =	*p*=0.153	*p*=0.271	
Triglycerides (mg/dl)	Pre	117.42 (72.85; 162.00)	123.81 (94.74; 152.88)	*p*=0.749
	Post	107.00 (65.57; 148.42)	124.18 (88.14; 160.21)	
	*p* =	***p*=0.004** ^**¥**^	*p*=0.966	

*Data are shown as mean and (95%CI). Groups are described as; (HIIT+RT) order session of high-intensity interval training plus resistance training, (RT+HIIT) order session of resistance training plus high-intensity interval training. (†) Between-group baseline comparisons were measured by General Lineal Model, using a Univariant test. Within-group comparisons by pre-post time were analyzed by Repeated Measures using 2-way ANOVA (Time×Group). (¥) Analyzed by non-parametric Wilcoxon test. Bold values denote significant differences at p≤0.05. All analyses were adjusted by age, height, and BMI*.

Comparing the HIIT+RT and RT+HIIT group with regard to delta (∆) changes from pre- to post-test, there were no significant differences between groups at ∆SBP, ∆DBP, ∆Tc, ∆LDL-c, ∆HDL-c, ∆Tg, and ∆FPG (Figure 2).

### Training-Induced Changes on Fitness

With regard to physical fitness outcomes, and considering absolute values, there were significant changes in the HIIT+RT group from pre- to post-training measurements in the 6Mwt from pre 499.28 (426.58; 571.98) vs. 637.14 (573.87; 700.40) m, *p*<0.0001 (Table 4). Similarly, in the RT+HIIT group, there were significant changes in this outcome from pre 533.63 (491.33; 575.94) vs. 597.27 (505.49; 689.04) m, *p*=0.048 (Table 4). No other significant pre- or post-training changes were detected in both groups at these parameters.

**Table 4:** Training-induced changes on strength and endurance performance in morbid obesity patients after 16weeks of concurrent training applied in two different concurrent training session modalities; CT as HIIT + RT, or CT applied as RT+HIIT.

Outcomes	Time	Groups		Baseline ^†^*p* value
		HIIT+RT	RT+HIIT	
**Strength/endurance performance**				
HGS (kg)	Pre	26.28 (22.51; 30.05)	29.72 (23.61; 35.83)	*p*=0.263
	Post	28.21 (23.60; 32.81)	31.18 (25.87; 36.48)	
	*p* =	*p*=0.090	*p*=0.249	
Ratio HGS/skeletal muscle mass	Pre	0.53 (0.47; 0.59)	0.57 (0.44; 0.71)	*p*=0.560
	Post	0.57 (0.49; 0.65)	0.61 (0.49; 0.74)	
	*p* =	*p*=0.076	*p*=0.295	
Ratio HGS/body mass	Pre	0.26 (0.23; 0.29)	0.28 (0.20; 0.35)	*p*=0.792
	Post	0.29 (0.22; 0.36)	0.30 (0.23; 0.37)	
	*p* =	***p*=0.045**	*p*=0.106	
Ratio HGS/lean mass	Pre	0.50 (0.44; 0.56)	0.54 (0.42; 0.67)	*p*=0.558
	Post	0.54 (0.47; 0.62)	0.58 (0.46; 0.70)	
	*p* =	*p*=0.075	*p*=0.131|	
6min walking test (m)	Pre	499.28 (426.58; 571.98)	533.63 (491.33; 575.94)	*p*=0.463
	Post	637.14 (573.87; 700.40)	597.27 (505.49; 689.04)	
	*p* =	***p*<0.0001**	***p*=0.048**	

*Data are shown as mean and (95%CI). Groups are described as: (HIIT+RT) order session of high-intensity interval training plus resistance training, (RT+HIIT) order session of resistance training plus high-intensity interval training. (HGS) Handgrip muscle strength. (†) Between-group baseline comparisons were measured by a General Lineal Model, using a Univariant test. Within-group comparisons by pre-post time were analyzed by Repeated Measures using 2-way ANOVA (Group×Time). Bold values denote significant differences at p≤0.05. All analyses were adjusted by age, height, and BMI*.

Comparing the HIIT+RT and RT+HIIT group in terms of delta (∆) changes from pre- to post-test, there were no significant differences between groups in ∆HGS, ∆HGS/BM ratio, ∆HGS/SMM ratio, ∆HGS/LM ratio, ∆HDL-c, and ∆6Mwt (Figure 3).

### Interindividual Variability on Mets Markers After HIIT+RT or RT+HIIT Session Orders of CT

There was a different prevalence of NRs in improving (i.e., decreasing) WC comparing HIIT+RT 2 (18.1%) vs. RT+HIIT group 5 (50.0%), *p*<0.0001 (Figure 4A). The two groups can be compared as follows: HIIT+RT vs. RT+HIIT group SBP 4 (27.2%) vs. 4 (40.0%; Figure 4B), DBP 8 (72.7%) vs. 7 (70.0%; Figure 4C), FPG 8 (72.7%) vs. 9 (90.0%; Figure 4D), HDL-c 7 (63.6%) vs. 8 (80.0%; Figure 4E), and Tg 7 (63.6%) vs. 8 (80.0%; Figure 4F), there were no significant differences in these outcomes.

## Discussion

The purpose of the present study was to describe the effects and the interindividual variability of 20weeks of HIIT+RT compared with another group doing RT+HIIT in a sample of women with severe/morbid obesity who were at risk of MetS. The main results of this study were that: (i) considering the MetS outcomes, the WC adiposity marker had significantly less prevalence of NRs in the HIIT+RT compared to the RT+HIIT order session after 20weeks of CT (Figure 4A) (ii) when both order session groups demonstrated significant improvements in MetS markers, such as at SBP (Figure 2A), or in secondary outcomes such as 6Mwt, for example (Figure 4E), there were no differences by group. Additionally, considering the overall training-induced changes for both HIIT+RT and RT+HIIT groups, a slight advantage in the RT+HIIT group was observed favouring more beneficial effects than the HIIT+RT group (Tables 2 and 3).

Although there are only a minor number of studies reporting the Rs and NRs phenomenon, there is little knowledge about this topic exploring exercise methodologies such as CT variations in clinical populations like the morbidly obese. Along these lines, and from our previous experience with obesity patients, for example, after an RT programme (20weeks, eight exercises), morbid obesity patients showed an NR prevalence of 42.8% compared with obesity patients (85.4%). Interestingly, however, morbid obesity patients showed higher benefits of decreasing WC ∆ –10.1 than their obese peers ∆ –2 to −6cm (Delgado-Floody et al., 2019). In this sense, the evidence shows the apparent role of the previously reported ‘health status’ factor in the prevalence of NRs, where populations of women with more disease clearly experience more benefits after exercise than peers with a minor degree of disease (Alvarez et al., 2017). However, one of the most intriguing results revealed in the present study regarding the minor prevalence of NRs for decreasing WC in the HIIT+RT 2 (18.1) vs. RT+HIIT 5 (50.0%; Figure 4A) is shown by contrast with the minor degree of reduction of WC in HIIT+RT ∆ –1.2 vs. RT+HIIT ∆ –2.7cm (Figure 1C). Although this difference is more than approximately twofold of WC reduction and was non-significant between groups, we could presume that independent of showing a higher prevalence of NRs for decreasing WC in the CT order session of RT+HIIT rather than the HIIT+RT group (Figure 4A), the training-induced changes are independent of the NRs phenomenon.

Additionally, a study conducted in overweight and obese subjects showed that 6weeks of HIIT (20-min protocol, consisting of 4min of cycling at 15% of maximum anaerobic power [Max-AP] followed by 30s at 85% of Max-AP) decreased BF% ∆ –0.88%, and BMI ∆ –0.26kg/m^2^ (Fisher et al., 2015). Another study by Alvarez et al. (2018) reported that 16weeks of HIIT (7–10×1min exercise with 2min of rest) reduced body mass ∆ −3.3kg, BMI ∆ −1.4% kg/m^2^, and BF% ∆ −5.8% in a prehypertensive and overweight/obese cohort of women.

Additionally, a 12-week RT programme (three times weekly, 60min/session, 17 strength exercises at 60–70% 1RM intensity) reported a significant reduction in both SBP ∆ –12.3mmHg, and DBP ∆ –11.2mmHg in obese men (Klimcakova et al., 2006). Although the authors have not reported the IVET as Rs and NRs, these blood pressure benefits are in accordance with the previous findings of our research team of ∆ –10.4mmHg at SBP, and ∆ –7.0mmHg at DBP, in morbidly obese patients after RT, where interestingly, these blood pressure benefits occurred independently of a weight loss (Delgado-Floody et al., 2019). Along similar lines, part of the exercise capacity for improving vasculature was the subject of a recent study conducted in young obese women which compared a HIIT group (4×4min at 85–95% of HR_max_, interspersed with 3-min periods of active recovery at 65–75% of HR_max_) with an MICT group (41min at 65–75% of HR_max_). This showed that both exercise protocols significantly reduced carotid–femoral pulse wave velocity by Δ−0.37 and Δ−0.35, respectively. Furthermore, significant reductions in brachial SBP Δ−6.3, and central SBP Δ−6.6mmHg were observed after HIIT (de Oliveira et al., 2020). Thus, the authors concluded that HIIT and MICT reduced arterial stiffness in obese young women, therefore showing salutary benefits as an antihypertensive nondrug therapy. Another study which investigated the effects of 6weeks of HIIT (10×1min intervals at 90–100% peak workload) or MICT (30min at 65–75% peak HR) on blood pressure and aortic stiffness in males who were overweight/obese, reported that HIIT was effective for reducing BP ∆ ~3–5mmHg, also there were MICT-induced moderate reductions in diastolic blood pressure (peripheral; ∆ −3.4mmHg and central; ∆ −3mmHg; Clark et al., 2020). From our previous experience, a 16week-HIIT programme (7–10×1min exercise with 2min of rest) reduced SBP Δ −8.0mmHg, and DBP Δ −5.8mmHg in sedentary and overweight/obese women (Alvarez et al., 2018). Thus, the mechanisms by which exercise training decreases blood pressure have been explained in part by a reduction of arterial stiffness, an improvement of endothelial mediated vasodilation, a reduction in vascular peripheral resistance, an increase of the novel peptide apelin by the nitric oxide plasma levels, and a decrease in sympathetic nervous activity (Alvarez et al., 2018) to name a few. Likewise, WC, SBP, and DBP are important parts of the MetS in which both HIIT and RT have been shown to benefit populations with higher adiposity.

In other secondary outcomes, only the RT+HIIT group reported changes in Tc (Table 3). In this sense, a 16week HIIT programme (7–10×1min exercise with 2min of rest) reduced Tc Δ −8mg/dl, LDL-c Δ −2.6mg/dl, Tg Δ −13.9mg/dl, and increased HDL-c Δ +5mg/dl in overweight/obese women (Alvarez et al., 2018). Following our experiences, similarly 20weeks of RT (three sessions/week, 4–8 exercise) using free weights reduced Tc Δ −7.5mg/dl in adults with obesity or morbid obesity (Delgado-Floody et al., 2019). Thus, at this level of adiposity, our present findings are in accordance with previous benefits of HIIT or RT in decreasing WC (a marker of visceral subcutaneous fat), as well as other fat sources such as the lipoproteins Tc, LDL-c, and Tg.

Both groups (HIIT+RT and RT+ HIIT) showed significant changes of the CRF marker 6Mwt, without a difference between the groups (Figure 2E). In this sense, for example, 34 sessions of HIIT (3–7 repetitions of 3min bouts of high-intensity walking [100% of$\dot{\;V}$O_2peak_], interspersed by 1.5min walking at low intensity) improved the$\dot{\;V}$O_2peak_ ∆+16% in obese subjects (Vaccari et al., 2020). Interestingly, RT has also been shown to improve CRF in some clinical populations. Following this, a study comparing 8weeks of RT (the programme started with two sets of 18–20 maximal repetitions and progressed to three sets of 10–14 maximal repetitions with a rest of 1–2min between sets) and CT (30min of MICT 40–70 HR reserve and 30min of RT per session) showed that the CRF was increased in RT ∆ +1.5ml/kg/min and CT ∆ +7.7ml/kg/min in obese adults (Schroeder et al., 2019). Considering that in the present study both HIIT+RT and RT+HIIT order session groups increased their performance in the 6Mt by ∆ +137.8 and ∆ +63.3m, respectively, (Figure 3E), together with the overall training-induced changes of RT at level of both MetS and secondary outcomes, we can presume that in a CT intervention for clinical populations, the RT scheme can play a major role in the health-related benefits of morbidly obese patients.

A limitation of this study was that the foods habits (diet) of the participants during the intervention were not measured. By contrast, a strength of this study was that we included not only MetS markers but also different outcomes such as anthropometric, body composition, strength, and CRF fitness markers, as well as highlighting the severely/morbidly obese clinical cohort with whom we developed our research project. Considering the expensive and long treatments before bariatric surgery, the topic of IVET is of high interest and value.

In conclusion, the HIIT+RT intervention promotes a smaller prevalence of NRs than the RT+HIIT order session of CT. Overall there is a slight advantage in the RT+HIIT group in favour of more beneficial training-induced effects compared to the HIIT+RT group.

## Data Availability Statement

The raw data supporting the conclusions of this article will be made available by the authors, without undue reservation.

## Ethics Statement

The studies involving human participants were reviewed and approved by the Ethical Committee of the Universidad de La Frontera, Temuco, Chile (ACTA N°071_18). The patients/participants provided their written informed consent to participate in this study.

## Author Contributions

PD-F and CA: conceptualization, formal analysis, and writing -review and editing. PD-F and CA: methodology. MR-P: software. CA: validation. CA and PD-F: formal analysis. PD-F, CA, and AS-M: investigation. PD-F, CA, AS-M, CM-S, CV, and FC-N: data curation. AS-M, MR-P, CV, and DJ-M: writing -original draft preparation. All authors have read and agreed to the published version of the manuscript.

## Funding

This research was funded by the Project FRO1895 and Project DI18-0043 by the University de La Frontera. CA was funded partially by Universidad de Los Lagos. AS-M was supported by the Spanish Ministry of Science, Innovation and Universities (ref. RTI2018â€“093302-A-I00).

## Conflict of Interest

The authors declare that the research was conducted in the absence of any commercial or financial relationships that could be construed as a potential conflict of interest.

## Publisher’s Note

All claims expressed in this article are solely those of the authors and do not necessarily represent those of their affiliated organizations, or those of the publisher, the editors and the reviewers. Any product that may be evaluated in this article, or claim that may be made by its manufacturer, is not guaranteed or endorsed by the publisher.

## Abbreviations

CT: Concurrent training
MetS: Metabolic syndrome
HIIT: High-intensity interval training
RT: Resistance training
WC: Waist circumference
SBP: Systolic blood pressure
DBP: Diastolic blood pressure
HDL-c: High-density lipoproteins
Tc: Total cholesterol
LDL-c: Low-density lipoproteins.
Tg: Triglycerides
FPG: Fasting plasma glucose
6Mwt: Six minutes walking test
IVET: Interindividual variability to exercise training
TE: Technical error of measurement
Rs: Responders
NRs: Nonresponders
MICT: Moderate-intensity continuous training
BMI: Body mass index
CRF: Cardiorespiratory fitness
HGS: Handgrip strength
SMM: Skeletal muscle mass
LM: Lean mass
