# Supplementary figures and images for: The Effects of Two Different Concurrent Training Configurations on Markers of Metabolic Syndrome and Fitness in Women With Severe/Morbid Obesity: A Randomised Controlled Trial
Source: Front Physiol. 2021 Sep 21;12:694798. doi: 10.3389/fphys.2021.694798 (PMC8491741; doi:10.3389/fphys.2021.694798)

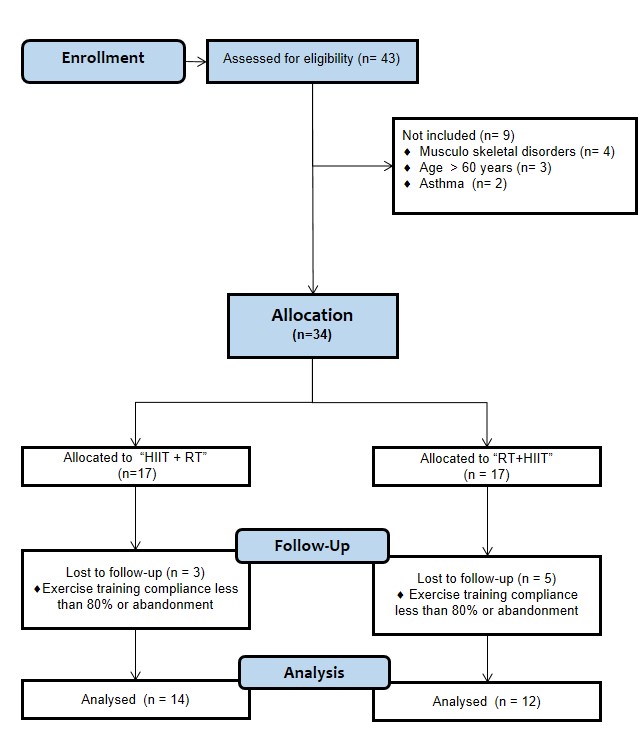

Supplement: Supplementary file 1 [file Image_1.JPEG]
